# Expression, purification and immobilization of tannase from *Staphylococcus lugdunensis* MTCC 3614

**DOI:** 10.1186/s13568-016-0261-5

**Published:** 2016-10-04

**Authors:** Amballa Chaitanyakumar, M. Anbalagan

**Affiliations:** School of Bio-Sciences and Technology, VIT University, Vellore, 632014 India

**Keywords:** Tannase, *S. lugdunensis*, Overexpression, *E. coli*

## Abstract

Enzymes find their applications in various industries, due to their error free conversion of substrate into product. Tannase is an enzyme used by various industries for degradation of tannin. Biochemical characterization of a specific enzyme from one organism to other is one of the ways to search for enzymes with better traits for industrial applications. Here, tannase encoding gene from *Staphylococcus lugdunensis* was cloned and suitability of the enzyme in various conditions was analysed to find its application in various industry. The recombinant protein was expressed with 6× His tag and purified using nickel affinity beads. The enzyme was purified up to homogeneity, with approximate molecular weight of 66 kDa. Purified tannase exhibited specific activity of about 716 U/mg. Optimum enzyme activity was found to be 40 °C at pH 7.0. Biochemical characterization revealed; metal ions such as Zn^2+^, Fe^2+^, Fe^3+^ and Mn^2+^ inhibited tannase activity, and SDS at lower concentration, increased tannase activity. Non polar organic solvents increased the tannase activity and polar solvents inhibited the tannase activity. Tannase immobilization studies show protection of the enzyme under wide range of pH and temperature. Also in this study we report a method for recovery and repeated use of the tannase.

## Introduction

Enzyme catalysed reactions are more preferred over chemical catalyst due to its several advantages; hence enzymes find a wide variety of applications in various industries (Gurung et al. 2013; Cherry and Fidantsef 2003). Microbes serve as a source for numerous enzymes with wide industrial applications (Demain 2000). Limitations in the quantity of enzyme produced by an organism compels, cloning and over expression of microbial enzymes regularly. Moreover, cloning of genes encoding enzymes of industrial importance is an essential step for engineering of enzymes for better traits. Comparison of an enzyme activity across different species (Taylor et al. 2002), screening of enzymes with random mutations (Cherry and Fidantsef 2003) and introduction of specific mutations in the enzymes (Neylon 2004; Cherry and Fidantsef 2003) are some of the methods to obtain enzymes for industrial use with desirable traits for various industrial applications.

Tannins are polyphenolic compounds produced by plants in order to protect themselves from invading microorganisms and herbivores (Buzzini et al. 2008). Tannin causes indigestion in herbivores when ingested; sometimes leading to death when ingested in more quantities (Butler 1992). Microbes produce tannase enzyme as a strategy to protect itself from tannin. Tannase enzyme (Tannin acyl hydrolase EC 3.1.1.20) hydrolyzes ester bonds in tannin to produce glucose and gallic acid.

Tannase is widely used in industries such as food, chemical, pharmaceuticals, breweries, tannery effluent treatment and production of animal feed (Aguilar et al. 2007). Tannase is also widely used for the production of gallic acid, which is a key intermediate required for the synthesis of an antibiotic drug, trimethoprim and used to produce propyl gallate, which is mainly used as an antioxidant in fats, oils and beverages (Miura et al. 2013). Gallic acid is also used in the fabrication of semiconductors, dyes and in photographic revelation (Chávez-González et al. 2012). Thus tannase finds its application in several industries.

Given its wide range of applications, there is a need for large scale production of this enzyme and more studies are required for production of good quality and quantity of tannase. Given the little amount of tannase produced by the micro-organisms, cloning and over expression of tannase gene is the feasible process to decrease production cost of the enzyme. Hatamoto et al. (1996) reported first cloning and expression of tannase from *Aspergillus oryzae*. Fungal tannase gene heterologously expressed in *Saccharomyces cerevisiae* and *Pichia pastoris* produced high amounts of recombinant tannase. Fungal tannase is made up of more than one subunit (Yao et al. 2014), which makes it difficult for over expression and purification. Compared to fungal tannase, bacterial tannase is made up of single subunit (Ren et al. 2013), which makes, cloning over expression and purification very easy.

Improvement of enzymes with desired industrial properties requires a complex and tedious methods of protein engineering or screening for mutant enzymes with desired activities (Neylon 2004; Cherry and Fidantsef 2003). Comparison and characterization of enzymes produced across different organisms, will give us an idea about choosing better enzymes with desired industrial properties. As mentioned before, tannase enzyme has been cloned and characterized in different organism, in the current study, for the first time, tannase gene from *S. lugdanensis* have been cloned, expressed, characterized and immobilized. The properties of the enzyme are compared with other bacterial tannase enzymes reported earlier. Also the immobilized enzyme was characterized suit its application in various industries.

## Materials and Methods

### Microorganisms

Bacterial strains *S. lugdunensis* MTCC3614 was acquired from MTCC (India), and cultured in Trypticase soya broth medium at 35 °C in a temperature controlled shaker at 150 rpm. For transformation and expression studies, *E. coli* DH5α and *E. coli* BL21 (DE3) were cultured in Luria–Bertani broth (LB) and incubated over-night at 37 °C.

### Cloning of tannase gene *tan*A

Genomic DNA was extracted from *S. lugdunensis* by CTAB NaCl method (Willner et al. 2012). Primers with the following sequences were synthesized in sigma-aldrich (Bangalore). SlF (gtacGGATCC*atgaaaaagactttc*) and SlR (ggccCTCGAG*ctattttttattaatac*) (additional nucleotides indicated in lower case, restriction sequence in higher case and the gene sequence in italics). A *Bam*HI restriction site was introduced in the forward primer (SlF) and to the reverse primer (SlR) *Xho*I restriction site was introduced. The *tan*A gene (NCBI accession number AB244239) along with flanking restriction sites was PCR amplified using the above mentioned primers along with genomic DNA isolated from *S. lugdunensis* as template. PCR conditions included an initial denaturation at 95 °C for 5 min, followed with 30 cycles of 95 °C for 1 min, 44 °C for 1 min, 68 °C for 2 min and final extension at 68 °C for 10 min. Prime STAR GXL DNA polymerase (TaKaRa) was used for reaction. Plasmid pET28a was obtained from Novagen company (Madison USA) and amplified in *E. coli* (DH5α). For plasmid isolation, Bio Basic plasmid isolation kit (India) was used. Restriction enzymes *Bam*HI and *Xho*I were obtained from NEB and NEB double digest standard protocols followed for restriction digestion of pET28a vector and the purified PCR product. After restriction digestion, pET28a and PCR product were purified on agarose gel using Bio-basic gel purification Kit. Gel purified pET28a and PCR products were mixed in the ratio of 1:3 and ligation reaction was carried out using TaKaRa ligation kit. The ligated product was transformed into chemically competent *E. coli* (DH5α) cells and plated on LB agar plate with 35 μg/ml of kanamycin. Single colonies from the plate was randomly picked and inoculated individually in liquid LB broth with kanamycin for plasmid isolation. Plasmids isolated from these clones were screened for presence of inserts by restriction digestion, using same restriction enzymes used for cloning. The authenticity of clone was tested by sequencing the insert and comparing the DNA sequence with NCBI data base.

### Protein expression and purification

Recombinant pET28a vector (referred as ptanET 28a) was transformed into *E. coli* (BL21DE3) and used for expression and purification of tannase enzyme. The *E. coli* (BL21 DE3) containing ptanET 28a vector was inoculated in LB broth with kanamycin and grown overnight in a 37 °C incubator shaker. Overnight culture was diluted with fresh medium to obtain a culture OD of 0.1 and the culture was grown until the OD value 0.6 was reached. For induction of protein from the plasmid, this culture was incubated with 1 mM isopropyl β-d-1-thiogalactopyranoside (IPTG) at room temperature (16 h). Culture without IPTG was grown under identical condition was used as control. At the end of incubation, cultures were centrifuged at 5000 rpm for 5 min at 4 °C, the supernatant was removed and pellet was further processed for protein purification. To the cell pellet, phosphate buffer (pH 7.0) was added and cells were lysed by sonication. Whole cell lysate were cleared by centrifuging at 10,000 rpm for 10 min at 4 °C, the soluble protein solution in the supernatant was further processed for recombinant protein purification.

Nickel NTA agarose resin was used for separating 6× His tagged recombinant tannase from the whole cell lysate. Nickel NTA resin was equilibrated for 1 h with binding buffer (50 mM NaH_2_PO_4_, 300 mM NaCl, 10 mM imidazole, pH 8.0). Buffer equilibrated resin (0.5 ml) was separated and added to the cell lysate. The suspension was mixed gently for 1 h at room temperature in order to allow binding of recombinant tannase to nickel NTA agarose. At the end of incubation period, the contents were centrifuged at 3000 rpm for 5 min to sediment the resin. The supernatant was carefully removed and pellet was washed thrice with ten volumes of wash buffer (50 mM NaH_2_PO_4_, 300 mM NaCl, 20 mM imidazole pH 8.0). After the last wash, the supernatant was removed and pure enzyme was eluted by adding one volume of elution buffer (50 mM NaH_2_PO_4_, 300 mM NaCl, 250 mM imidazole pH 8.0) and mixed thoroughly for 10 min at room temperature. At the end of incubation time, mixture was centrifuged at 3000 rpm for 5 min. The supernatant were collected and transferred to fresh tubes and stored at 4 °C. Purity of the enzyme was determined by sodium dodecyl sulfate polyacrylamide gel electrophoresis (SDS-PAGE) analysis.

### Enzyme activity

Tannase enzyme activity was determined by rhodanine method (Sharma et al. 2000). Rhodanine specifically reacts with gallic acid to form chromogen but not other phenolic compounds. Tannase activity was calculated based on the liberation of gallic acid in specific enzymatic assay conditions. Solution containing 50 μl of enzyme was incubated with 100 μl of substrate methyl gallate (0.01 M concentration) for 5 min at 37 °C. At the end of incubation time, the reaction was stopped by adding 300 μl methanolic rhodanine (0.667 %) and the tubes were incubated for 3 min at room temperature. To all tubes 100 μl of 0.5 M KOH and water is added to a final volume of 2 ml before taking reading at 520 nm. Control tubes were incubated with same concentration of heat denatured enzyme along with substrate and methanolic rhodanine, blank was made without enzyme. Amount of gallic acid liberated was estimated using standard gallic acid calibration curve. One unit of tannase was taken as the amount of enzyme required for liberating one µmol of gallic acid per ml per min. Amount of protein present in the sample was estimated using protocol of Bradford with BSA as standard 1 mg/ml.

### Immobilization

Crude tannase (2000 Units/ml) was slowly mixed with 10 ml of sodium alginate (5 %) by avoiding air bubbles. The tannase sodium alginate mixture was added drop by drop into ice cold 0.7 % CaCl_2_ solution with continuous stirring. The beads thus formed were transferred to fresh 0.7 % CaCl_2_ solution and stored for 2 h at 4 °C to strengthen the beads. The beads were then washed with 0.2 mm phosphate buffer (pH 7.0) for removing any unbound protein. The immobilization yield was determined by dividing immobilized beads specific activity by total enzyme specific activity used for immobilization. The beads were then stored at 4 °C for further analysis.

### Effect of temperature

The effect of temperature on tannase activity was studied for crude, purified and immobilized enzyme at different temperatures (4, 10, 20, 30, 40, 50, 60 and 70 °C). The enzymatic reaction mixture was incubated for 5 min in the above mentioned temperatures and the enzyme activity was measured as mentioned above.

### Effect of pH

Effect of pH on tannase activity was studied by incubating crude, purified and immobilized enzymes at different pH (3.0–9.0) and constant temperature 40 °C for 5 min. pH study was performed using buffers such as 0.02 M citrate buffer (pH 3.0–5.0), 0.02 M phosphate buffer (pH 6.0–7.0) and 0.02 M tris buffer (pH 8.0–9.0).

### Effects of additives

Effect of additives on tannase activity was studied by incubating purified tannase with 1 % of different additives such as Tween20, Tween80, TritonX 100, β-mercaptoethanol, sodium dodecyl sulfate (SDS) and ethylenediaminetetraacetic acid (EDTA) for 30 min at room temperature. The residual tannase was collected for estimation of enzyme activity. Purified tannase without additives was kept as control and its activity was taken as 100 % activity and based on this relative activity of tannase with different additives were calculated.

### Effects of metal ions

Effects of metal ions on tannase activity was determined by incubating purified tannase with metal ions such as MgCl_2_, CaCl_2_, NaCl_2_, ZnCl_2_, FeCl_2_, MnCl_2_, KCl and FeCl_3_ for 30 min at room temperature for three different concentrations (1, 5 and 10 mM). After incubation residual tannase was collected and used for determination of tannase activity. Purified tannase activity in the absence of any metal ions was taken as control and its relative activity was considered 100 % which was used to calculate relative activity of the tannase with different metal ions.

### Effects of organic solvents

Purified tannase was pre-incubated with different concentrations (20, 40 and 60 %) of organic solvents such as methanol, dimethyl sulfoxide (DMSO), hexane, isoamyl alcohol, toluene, butanol and benzene for 30 min at room temperature to determine the effects of organic solvents on the tannase. Purified tannase in the absence of any organic solvent was taken as control and its relative activity as considered 100 % activity of tannase and calculated tannase relative activity after incubated with organic solvents.

### Reusability of immobilized enzyme

The reusability of immobilized tannase on calcium alginate beads was studied by incubation of 0.5 g of immobilized beads with 1.5 ml of 0.01 M methyl gallate and 1.5 ml of phosphate buffer (pH 7.0) for 30 min at 40 °C. Activity was estimated by using methyl rhodanine method. The same gel beads were then washed with phosphate buffer and re-incubated with another substrate solution; this procedure was repeated for 15 times, and the initial activity of the enzyme was considered as 100 %. The relative activity was expressed as a percentage of the starting operational activity.

## Results

### Cloning and expression of tannase

**Fig. 1:**
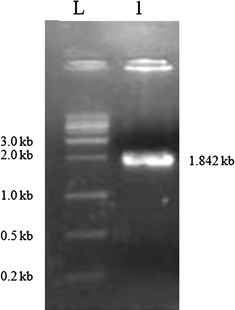
PCR amplification of tannase gene from *S. lugdunensis*. PCR was performed with primers having homology with tannase gene of *S. lugdunensis*. The PCR product was separated on 1 % agarose (*lane 2*) along with DNA marker (*lane 1*)

**Fig. 2:**
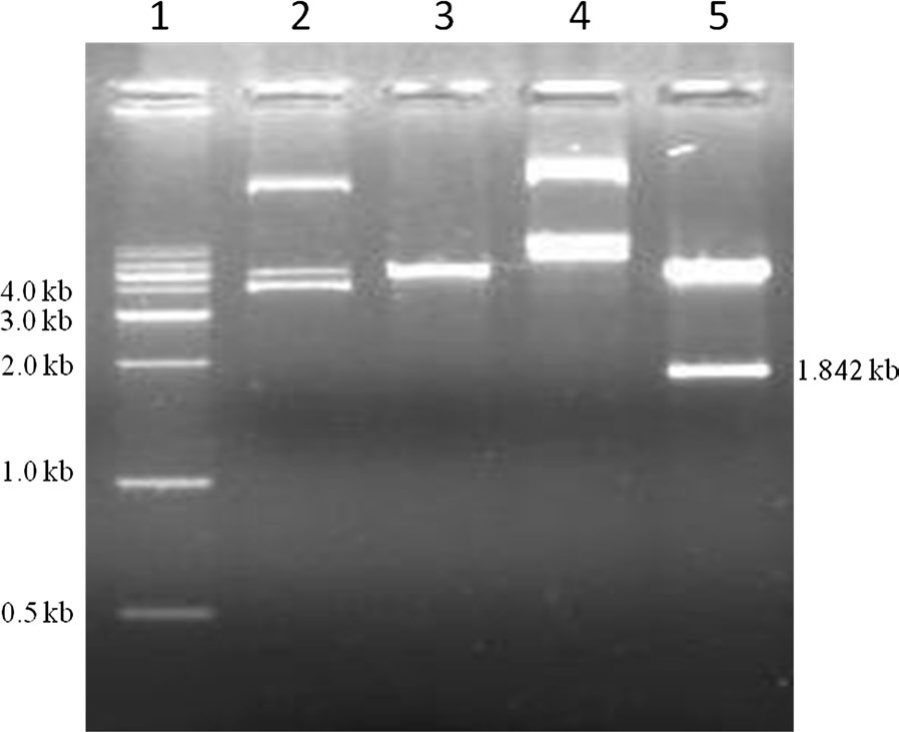
Release of insert from pET 28a vector with restriction enzymes. Recombinant vector and empty vector were incubated with or without restriction enzymes used for cloning for 2 h at 37 °C. The products were separated on 1 % agarose gel. *Lane 1*: 1 kb marker; *Lane 2*: pET28a vector without enzymes; *Lane 3*: pET28a vector with restriction enzymes; *Lane 4*: Recombinant pET28a without restriction digestion; *Lane 5*: Recombinant pET28a vector with restriction digestion

Tannase being an important commercial enzyme, more variants of enzyme from microbial sources need to be characterized. In order to characterize tannase from *S. lugdunensis,* tannase encoding gene from *S. lugdunensis* was PCR amplified from its genomic DNA using specific primers. Separation of PCR product on agarose gel electrophoresis revealed a fragment of 1.8 kb size as shown in the Fig. 1. Tannase gene ORF was cloned into *Bam*HI and *Xho*I site of pET 28a vector multiple cloning site. Presence of tannase gene in the vector was confirmed by a) Release of cloned ORF with *Bam*HI and *Xho*I b) Sequencing the insert in the recombinant vector. As shown in Fig. 2, digestion of recombinant plasmid with *Bam*HI and *Xho*I released the insert which was absent in control vector. DNA sequencing and comparison of sequences with reference sequence in NCBI database revealed a 100 % match.

### Induction and protein purification

**Fig. 3:**
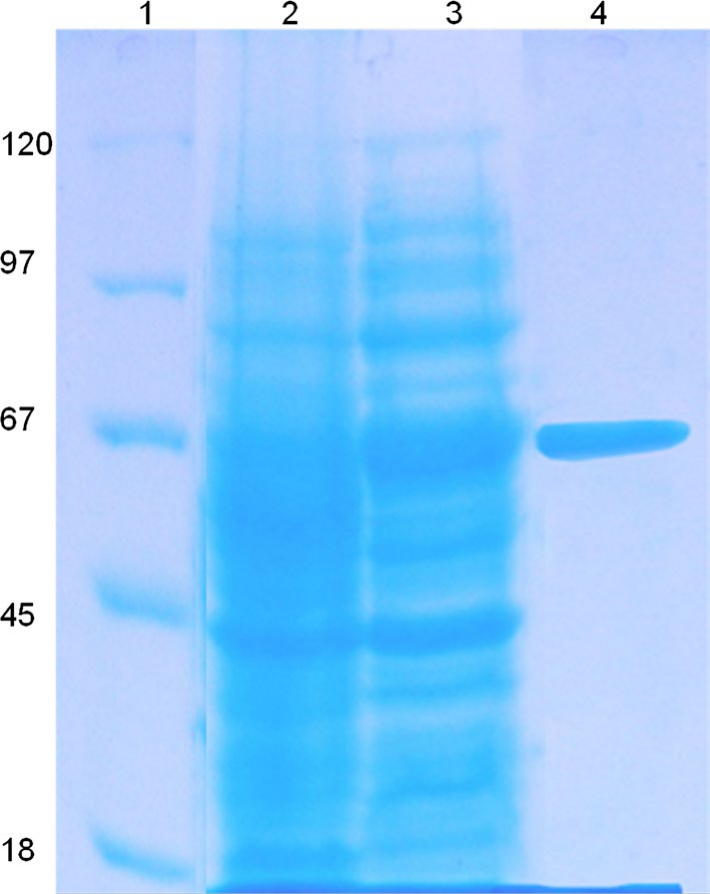
SDS- PAGE analysis of protein samples from bacteria induced with or without IPTG and purified Tannase protein. *Lane1*: Molecular weight marker, *Lane 2*: Soluble cell extracts from control bacteria, *Lane 3*. Cell lysate from IPTG induced bacteria and *lane 4*. Ni–NTA purified protein from IPTG induced bacterial whole cell lysate

After confirming the presence of tannase gene ORF in correct reading frame with 6× His sequence of the pET 28a expression vector, expression studies were carried out with ptanET 28a in BL-21 (DE3) strain. BL-21 bacteria carrying the plasmid was induced with IPTG for 16 h and protein profile was compared with the un-induced Bl-21 bacteria, and tannase with 6× His tag was purified from whole cell lysate using Ni NTA beads. As shown in the Fig. 3 protein samples from induced and un-induced BL-21 bacteria and purified tannase was separated on SDS-PAGE and stained with Coomassie blue. Ni NTA purification yielded 100 % pure protein as shown in the gel as a single band of molecular weight 66 kDa. The size of the band matched with the induced tannase in the whole cell lysate of BL-21.

### Effect of temperature

**Fig. 4:**
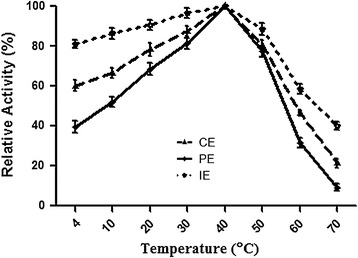
Effect of temperature on tannase activity of *S. lugdunensis*. Enzyme activity at different temperature was measured using *CE* crude enzyme, *PE* purified enzyme and *IE* immobilized enzyme. Values show relative levels at optimum temperature (40 °C)

The activity of tannase in (1) crude (2) purified and (3) immobilized tannase was tested and results are shown in Fig. 4. Highest tannase activity was found to be 40 °C for the three forms of tannase. However at lower temperature (4 °C) immobilized tannase showed highest activity (80 %) followed by crude (60 %) and purified tannase (40 %). The same trend was observed at higher temperature (70 °C); immobilized (39 %), crude (10 %) and no detectable activity with purified tannase.

### Effect of pH

**Fig. 5:**
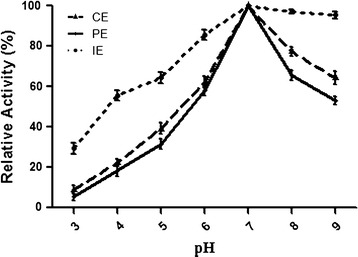
Effect of pH on tannase activity of *S. lugdunensis*. Enzyme activity at different pH was measured using *CE* crude enzyme, *PE* purified enzyme and *IE* immobilized enzyme. Values show relative levels at optimum pH (7.0)

At 40 °C effect of pH on tannase activity was measured for (1) crude (2) purified and (3) immobilized tannase and results are shown in Fig. 5. At pH 7.0 highest activity was found for the three forms of tannase. At extreme condition (both high and low pH) immobilized tannase showed better activity compare with crude and purified tannase. At pH 3.0, immobilized tannase activity 29 % and pH 9.0 immobilized tannase activity 95 % was observed.

### Effects of additives

**Fig. 6:**
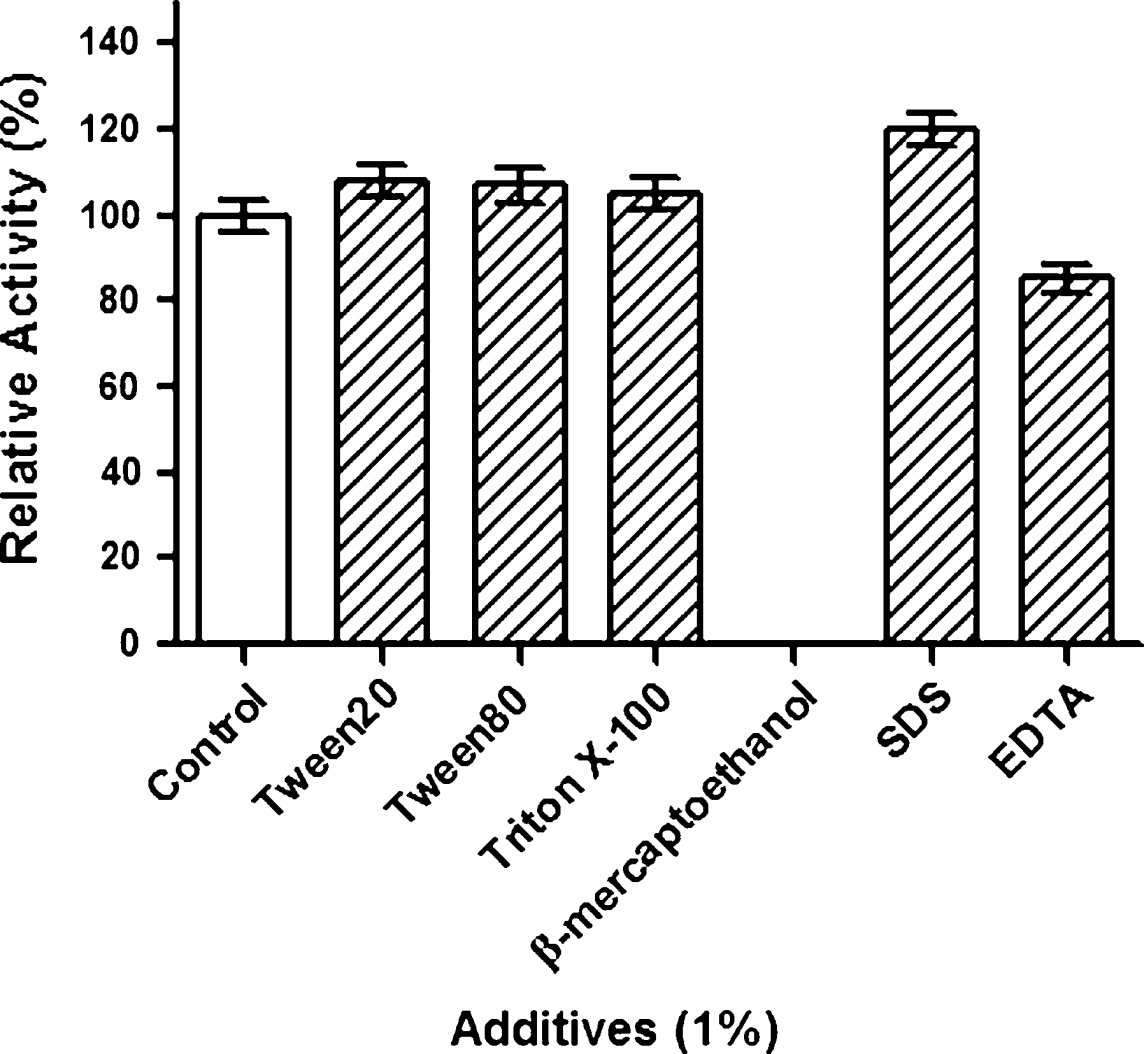
Effect of pre-incubation of detergents on *S. lugdunensis* tannase activity. Purified tannase was incubated for 30 min with or without 1 % of above mentioned detergents and enzyme assay was carried out. Values represent relative to control

Effects of different additives on tannase activity were monitored under optimum temperature and pH. Out of different detergents included in the study as listed in Fig. 6. Only SDS at concentration of 1 % increased 20 % of the tannase activity. At 1 % concentration EDTA shows slight inhibition (15 %) and β-mercaptoethanol completely inhibited tannase activity at 1 % concentration and other additive did not show any significant changes.

### Effect of metal ions

**Fig. 7:**
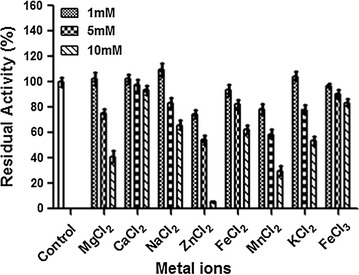
Effect of metal ions on *S. lugdunensis* tannase activity. Tannase was pre-incubated with or without different concentrations (1, 5, 10 mM) of metal ions and the enzyme activity was measured. Values indicate relative to control

Since several enzymes uses metal ions as co-factors, influence of several metal ions on tannase activity was tested at optimum temperature and pH (Fig. 7). Out of several metal ions tested, Na^+^ ion increased the enzyme activity by 10 % at 1 mM concentration, generally inhibition of tannase activity was observed in presence of high concentration of ions (except for Ca^2+^ ions).

### Effect of organic solvents

**Fig. 8:**
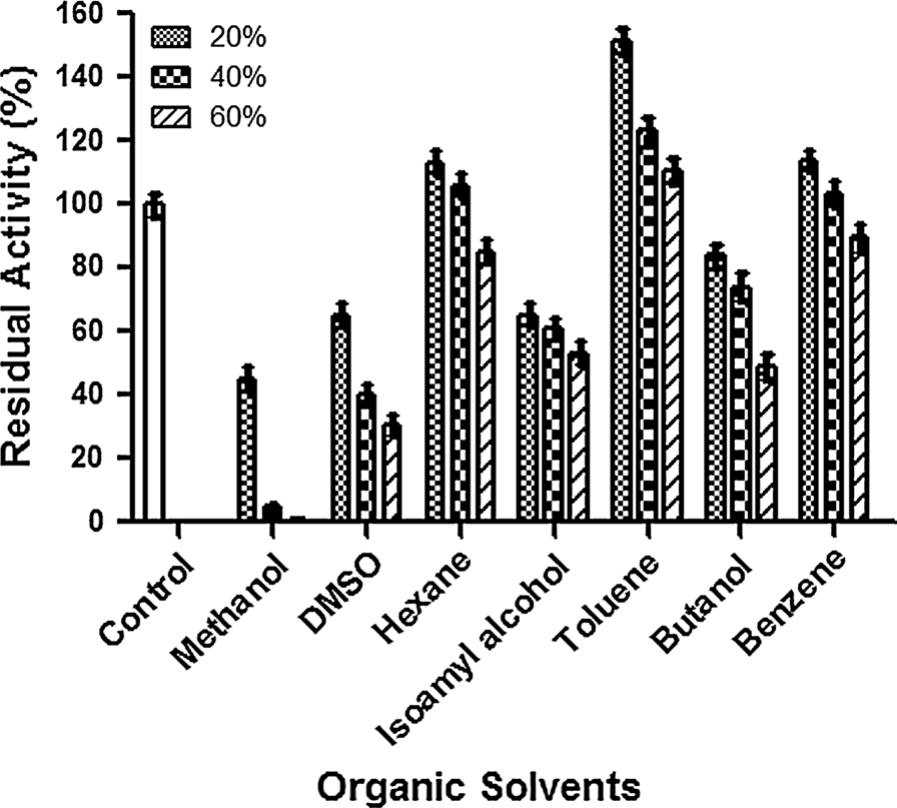
Effect of organic solvents on *S. lugdunensis* tannase activity. Tannase was pre-incubated for 30 min at room temperature with different percentage of below given list of organic solvents and activity of the enzyme was measured. Values indicate relative to control values

Effect of organic solvents on tannase activity was measured. The tannase activity was tested, in presence of various organic solvents in different concentrations Fig. 8. Generally non-polar solvents (Hexane, Toluene, and Benzene) increased the tannase activity and polar solvents (Methanol, DMSO, Isoamyl alcohol and Butanol) decreased the tannase activity. Toluene at 20 % concentration enhanced tannase activity by 50 %, hexane and benzene at 20 % concentration increased enzyme activity 15 %. DMSO and iso-amylalcohol at 20 % could inhibit the tannase by 35 % of activity; and methanol at same concentration shows 56 % inhibition of tannase activity.

### Stability and reusability of immobilized tannase

**Fig. 9:**
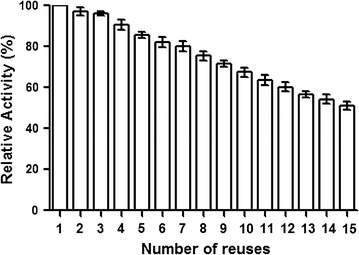
Relative activity of re-used immobilized tannase. Immobilized tannase beads were incubated with substrate and enzyme assay was carried out. At the end of incubation period, beads were recovered and re incubated with the substrate and enzyme activity was measured. Numbers indicate, number of times the same beads were used in the assay

Immobilized tannase reusability and stability at operational conditions were studied up to 15 cycles. Immobilized beads showed 80 % activity up to seven cycles, more than 50 % activity was observed even after 15 cycles Fig. 9.

## Discussion

Microbes produce a variety of enzymes to cater their metabolic needs under different physiological conditions. Tannase is one such enzyme produced by microbes to protect themselves from tannin mediated toxicity and to generate glucose from tannin under scarcity of glucose in the surrounding environment (Jiménez et al. 2014b). Tannase is expressed in the microbes only when it is required (Jana et al. 2014; Kumar et al. 2015), therefore, the tannase produced by the microorganisms are so little, that it cannot cater the needs of industries. Hence, cloning and expression of tannase is the way to produce large quantity of tannase. In this regard several studies have reported cloning and expression of tannase from different microorganisms. Bacterial tannase gene was cloned expressed and characterized from *Lactobacillus plantarum*, (Iwamoto et al. 2008), (Curiel et al. 2009) and (Ueda et al. 2014) *Streptococcus gallolyticus* (Jiménez et al. 2014a) and *Enterobacter* sp. (Prakash and John 2011).

Given the wide application of tannase in different industries, different type of tannase that can work at wide range of physical parameters such as temperature, pH, ionic strength, presence of detergent and organic solvents needs to be identified. Tannase like any other enzyme is made up of amino acids, the amino acid composition and number will determine the performance of the enzyme at the above mentioned parameters. Though several tannase encoding gene has been characterized before, tannase enzyme from *S. lugdunensis* has not been characterized so far. Given the reason that tannase encoded by *S. lugdunensis* differ in the number and composition of amino acids with tannase produced by other organisms (Noguchi et al. 2007). In this study for the first time we have characterized tannase from *S. lugdunensis.*

As mentioned before, bacterial tannase gene was cloned expressed and characterized from different bacterial species, in order to study if tannase from *S. lugdunensis* has any advantages with respect to industrial applications, tannase encoding gene was cloned, over expressed, characterized and immobilization studies were carried out. *S. lugdunensis* genome encodes tannase gene of size 1842 base pairs, genomic DNA PCR with primer pairs annealing to tannase gene of *S. lugdunensis* gave a PCR product of size 1.8 kb indicating PCR amplification of tannase encoding gene from the bacteria (Fig. 1). This PCR product was cloned into pET28a vector. Compared to control vector, release of 1.8 kb product from ptanET28a recombinant vector using restriction enzymes were used to clone the PCR product into the vector (Fig. 2), proves the construction of recombinant expression vector for tannase. Sequencing and alignment of insert sequence with NCBI data base revealed; the insert shared 100 % homology with tannase gene of *S. lugdunensis*.

Comparison of whole cell lysate obtained from IPTG induced and un-induced bacterial culture on SDS-PAGE, revealed a prominent band of size 66 kDa present only in IPTG induced samples (Fig. 3). In silico translation of *S. lugdunensis* tannase gene produced a 66 kDa protein and bacteria with ptanET28a produced identical 66 kDa protein, confirming our recombinant vector express tannase gene from *S. lugdunensis*. In this regard Noguchi et al. (2007) also reported *S. lugdunensis* encodes tannase enzyme with molecular weight of 66 kDa.

In order to detect tannase activity and for characterization of the enzyme, rhodanine assay (Sharma et al. 2000) was used. Since pET28a is designed to express the recombinant protein as a 6× His tagged version, the over expressed tannase was purified using Ni NTA beads, up to the purity of no detectable impurities was present on SDS-PAGE stained with Coommassie blue stain (Fig. 3). Purified form of tannase was used in all enzymatic assays involving characterization of this enzyme with a specific activity of about 716 U/mg. This was higher than other reported studies (577 U/mg) (Jiménez et al. 2014a), (84.3 U/mg) (Iwamoto et al. 2008) and (13.63 U/mg) (Prakash and John 2011).

Most of the bacterial tannase exhibit an optimal temperature in the range between 30 and 40 °C (Chávez-González et al. 2012). *S. lugdunensis* recombinant enzyme (crude, purified and immobilized tannase forms) showed similar optimal temperature of about 40 °C (Fig. 4). Studies conducted to identify maximum and minimum tolerant temperature for the tannase revealed; immobilized tannase showed 90 % activity at 50 °C, crude tannase showed 80 % activity and purified tannase gave 75 % activity at the same temperature. Similarly at 4 °C, immobilized tannase shows 80 % activity, crude and purified tannase showed 60 and 40 % activity respectively (Fig. 4). These results suggest that immobilization of tannase protects the tannase activity at both above and below optimal temperature. Previous reports also suggest that immobilization of tannase protects the enzyme activity at both higher and lower than optimal temperature (Iyer and Ananthanarayan 2008). These observations suggest that immobilization of tannase can find its applications in industries such as juice production, wine production, and instantaneous tea production requiring clarification of tannin at lower temperatures. In relation to this it has been reported that immobilization process significantly improved thermo stability of the immobilized enzyme (Chávez-González et al. 2012).

The tannase showed best activity at pH 7.0. Comparison of activity of tannase with crude, purefied and immobilized tannase, demonstrated that immobilized tannase performed better at wide range of (pH 4.0–9.0). Crude tannase showed 40 % activity at pH 5.0 and purified tannase at the same pH showed 30 % activity; whereas immobilized tannase exhibited 65 % activity at pH 5.0. Similarly there was not much effect of pH on the immobilized enzyme between pH 7.0–9.0 (Fig. 5) under same condition the activity of crude and purified tannase dropped drastically. During immobilization process, enzymes are covalently bonded with the matrix or encapsulated with the porous matrix, which resulted in less micro environmental exposure for the enzyme when compared to the free enzyme. Hence, pH changes in the solution exhibit less impact to the immobilized enzyme activity due to improper environmental exposure; when compared to the free enzyme. This data is being supported by similar observations in β-Glucosidase immobilization studies (Tu et al. 2006; Figueira et al. 2011).

Anionic detergents such as SDS are known to increase residual activity of enzyme due to its ability to create negative charge in the active site of the enzyme. Purified tannase from *S. lugdunensis* exhibited 21 % higher residual activity when compared with the controls (Fig. 6). Activity of enzymes that use metal ions as cofactors are drastically decreased in presence of EDTA, there was no drastic inhibition of *S. lugdunensis* tannase activity in presence of EDTA, suggesting this enzyme doesn’t use metal ions as co-factor. In related to this tannase activity was not influenced in presence of common metal ions (Fig. 7). However at high concentration of metal ions, the tannase activity was decreased due to increase in ionic strength of the solution, which might affect the tannase native structure. Since tannase belong to serine group of enzymes (Mizuno et al. 2014), β-mercaptoethanol completely abolished the tannase activity (Fig. 6). Inhibition of tannase activity by β-mercaptoethanol has been reported earlier (Yao et al. 2014; Jiménez et al. 2014a).

Organic solvents play a major role in food industries. These solvents are useful to dissolve substrate completely and also some time they are known to enhance the activity of enzymes by modifying their structure in non-polar environment (Sharma and Kanwar 2014; Nie et al. 2016). In our studies, at 20 %, non -polar solvents such as hexane, toluene and benzene significantly, increased the activity of S. *lugdanensis* tannase (Fig. 8). However polar solvent such as methanol, DMSO, iosamyl alcohol and butanol inhibited the enzyme activity. It has been previously reported that polar compounds decrease the water content in and around the enzyme micro environment that could decrease the enzyme activity as observed by us (Nie et al. 2016). The ability of polar solvents to inhibit tannase activity and non polar solvents to enhance tannase activity are reported in *Aspergillus awamori* (Chhokar et al. 2010), *Aspergillus carbonarius* (Valera et al. 2015), *Emericella nidulans,* (Gonçalves et al. 2011) and in *Enterobacter cloacae* (Beniwal et al. 2010).

The reuse number of immobilized enzymes is standout amongst the most vital perspectives for industrial application. An increased stability could make the immobilized enzyme more advantageous than its free form (Huang et al. 2008; Gupta et al. 2014). Recombinant tannase immobilized with calcium alginates beads and its reusable activity 80 % after six successful reuses and 50 % activity after fifteenth cycle (Fig. 9). After each successful cycle, loss of protein from immobilized beads was tested by estimating the protein from supernatant by Bradford method and no significant protein was found in it. Based on this result, it is assumed that loss of activity occurs due to inactivation of enzyme caused by the denaturation (Gupta et al. 2014; Ye et al. 2006).

Tannase enzyme finds its applications in wide variety of industries and also in treatment of effluent from industries. For the first time, tannase from *S. lugdunensis*, has been cloned, over expressed and characterized in this study. This study also provides data for wide range application of tannase under different physiological conditions. More over immobilization studies demonstrate the recovery and re use of the enzyme which will reduce the cost of production. The potential of using the immobilized tannase in various industries and industrial effluent are in progress.

## Abbreviations

ptanET 28a: recombinant pET 28a
IPTG: isopropyl β-d-1-thiogalactopyranoside
SDS-PAGE: sodium dodecyl sulfate polyacrylamide gel electrophoresis
SDS: sodium dodecyl sulfate
EDTA: ethylenediaminetetraacetic acid
DMSO: dimethyl sulfoxide
CE: crude enzyme
PE: purified enzyme
IE: immobilized enzyme
